# Interface-Engineered, Low-Damage IGZO/HfO_2_ Charge-Trapping Memory Devices Fabricated Using a Remote Plasma ALD Process

**DOI:** 10.3390/mi17060743

**Published:** 2026-06-19

**Authors:** Inkook Hwang, Hyeonwu Nam, Jiwon Kim, Byungwook Kim, Yongwoon Jang, Wookyung Lee, Minkyun Kang, Changbun Yoon

**Affiliations:** Department of Advanced Materials Engineering, Tech University of Korea, Siheung-si 15073, Republic of Korea; inkook@tukorea.ac.kr (I.H.); hyeonwu0604@tukorea.ac.kr (H.N.); jyw5414@tukorea.ac.kr (Y.J.);

**Keywords:** charge-trapping memory (CTM), IGZO (In–Ga–Zn–O), HfO_2_, atomic layer deposition (ALD), remote plasma (RP), crystallization, annealing temperature, memory window

## Abstract

In this study, charge-trapping memory (CTM) transistors were developed using indium gallium zinc oxide (IGZO) as the oxide semiconductor channel and high-k HfO_2_ as the charge-trapping layer, aiming for next-generation nonvolatile memory applications. To evaluate the impact of plasma exposure on film quality and device performance, HfO_2_ thin films were deposited via atomic layer deposition (ALD) using both direct plasma (DP) and remote plasma (RP) modes. Post-deposition annealing (PDA) was applied to the IGZO and HfO_2_ layers, with experiments conducted at various annealing temperatures to enhance the interfacial stability between the HfO_2_ layer and the IGZO channel. Electrical characterization results demonstrated that the RP-processed devices exhibited a wider memory window, reduced gate leakage current, and improved threshold voltage stability compared with the DP-processed devices. Thermal treatment effectively reduced the interfacial defect density and enhanced the crystallinity at the dielectric–channel interface. These findings underscore that the selection of the plasma process and annealing conditions is critical in determining the electrical characteristics and reliability of oxide semiconductor-based CTM devices.

## 1. Introduction

The increasing integration of artificial intelligence (AI) into modern electronic systems—including autonomous vehicles, smart devices, wearables, and Internet of Things (IoT) platforms—has driven the demand for high-density, low-power, and fast-operating memory technologies with long-term data retention capabilities [1,2,3,4,5]. These next-generation applications require memory devices that can simultaneously support continuous scaling, low energy consumption, and stable endurance. However, conventional Si-based flash memory technologies are approaching their physical and electrical limits [6,7,8,9,10,11]. As device dimensions continue to shrink into the deep sub-10 nm region, several critical issues arise: the exacerbation of the short channel effect (SCE), including drain-induced barrier lowering and gate-induced drain leakage, increased gate leakage due to thinner dielectric layers, and poor interface reliability. These challenges have prompted the exploration of novel materials and memory architectures to replace or augment the conventional charge storage mechanisms employed in flash memory devices [12,13,14,15,16].

Among various alternatives, charge-trapping memory (CTM) devices that utilize high-***k*** dielectric materials as charge storage layers have emerged as potential candidates for nonvolatile memory (NVM) applications. In particular, hafnium oxide (HfO_2_) has gained considerable attention owing to its wide bandgap (5.5–6.0 eV), high dielectric constant (20–25), high thermal and chemical stabilities, and compatibility with complementary metal–oxide–semiconductor (CMOS) processes. HfO_2_ inherently accommodates oxygen vacancies, which serve as trap sites for charge storage and enable fast program/erase (P/E) operations. Recent studies have further demonstrated that the crystallographic phase of HfO_2_ can be engineered to exhibit ferroelectric or perovskite-like behavior, opening new pathways for multilevel cell operation and low-voltage switching, which are essential in emerging applications such as neuromorphic computing, embedded NVM, and in-memory computing [17,18,19,20,21,22,23,24].

Parallel to the development of high-***k*** dielectrics, the need for improved channel materials has become increasingly important. As conventional Si channels experience mobility degradation and enhanced surface scattering under continuous scaling, metal oxide semiconductors have emerged as viable alternatives. In particular, indium gallium zinc oxide (IGZO) has shown promise as a next-generation channel material owing to its high electron mobility, wide bandgap, low off-state leakage current, and favorable interface characteristics [25,26,27,28,29]. These advantages make IGZO a suitable candidate for advanced low-power and high-performance NVM devices [30,31,32,33,34,35].

A key factor in achieving reliable CTM operation lies in the quality of the interface formed between the charge-trapping layer and the semiconductor channel. Interface defects, interfacial oxides, and plasma-induced damage can degrade charge transport, reduce threshold voltage stability, and shorten data retention times. Therefore, careful control over thin-film deposition processes is essential. Among various deposition techniques, atomic layer deposition (ALD) is a widely adopted method for fabricating ultra-thin, conformal dielectric layers in advanced semiconductor devices [36,37,38,39].

ALD offers atomic-scale thickness control and uniform step coverage in high-aspect-ratio structures, making it advantageous for emerging 3D memory architectures such as 3D-NAND and vertical NVM arrays. Plasma-enhanced ALD has been widely adopted for high-***k*** film deposition at low temperatures, as it can provide a high film density and a relatively high growth rate. However, the use of direct plasma (DP) ALD introduces energetic ions that can physically bombard the substrate surface, generating interface traps, inducing sub-gap states, and degrading the channel–dielectric interface. These adverse effects become particularly pronounced in ultrathin films and multi-layer stacks, limiting the scalability and reliability of DP-ALD processes [40,41,42,43,44]. Consequently, remote plasma ALD (RP-ALD) has emerged as a potential alternative. In RP-ALD, plasma is generated in a region separate from the deposition chamber, and only neutral radicals are introduced into the process area. This separation suppresses ion-induced damage and directional bombardment while still enabling effective precursor activation and stable film growth [45,46,47,48,49].

In this study, CTM transistors were fabricated using IGZO as the channel layer and HfO_2_ as the charge-trapping layer. To investigate the impact of plasma-induced damage on device performance, HfO_2_ thin films were deposited using both DP-ALD and RP-ALD processes. The IGZO channel layer was deposited via RF magnetron sputtering, after which both layers were simultaneously subjected to post-deposition annealing (PDA) at various temperatures to observe the resulting interfacial reactions. The electrical characteristics of the devices, including memory window, threshold voltage stability, and leakage current behavior, were evaluated and compared. Further, structural and chemical analyses, such as X-ray diffraction (XRD), X-ray photoelectron spectroscopy (XPS), and transmission electron microscopy (TEM), were conducted to examine possible differences in film crystallinity and interface quality between the two ALD methods. The advantages of RP-ALD over DP-ALD in terms of suppressing plasma-induced interface degradation and enhancing charge-trapping efficiency were evaluated. We demonstrate how RP-ALD enables more stable and reliable CTM operation, making it an effective deposition technique for fabricating advanced oxide semiconductor-based NVM devices [50,51,52,53,54].

## 2. Materials and Methods

### 2.1. CTM Device Fabrication Using IGZO and HfO_2_

**Figure 1:**
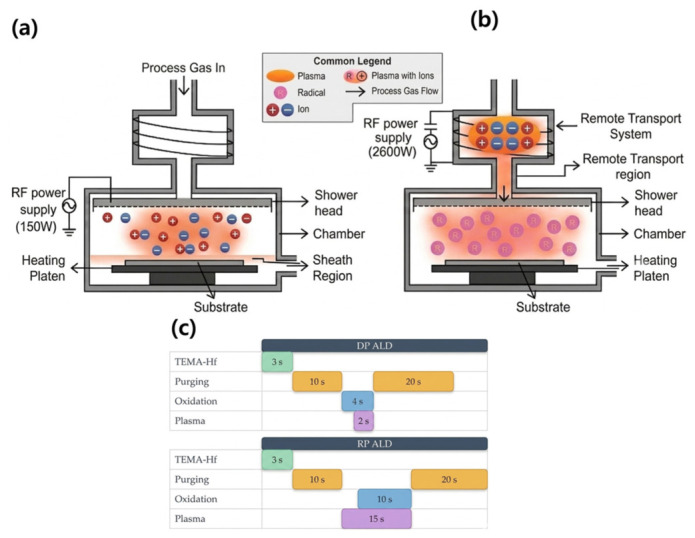
Schematic configurations of (**a**) direct plasma (DP) and (**b**) remote plasma (RP) ALD systems, and (**c**) ALD valve sequences and exposure times utilized for DP and RP ALD processes.

Figure 1 outlines the experimental setups and process timing sequences used in this study. Schematic diagrams of the DP-ALD and RP-ALD systems are presented in Figure 1a,b, while the corresponding valve switching sequences are shown in Figure 1c. Unlike DP-ALD, in which the substrate is directly exposed to the plasma discharge, RP-ALD spatially separates the plasma generation region from the main chamber using a remote inductively coupled plasma source and an Ar carrier gas. This dual-mode capability was integrated into a unified ALD system to isolate the distinct effects of the plasma mechanisms while maintaining identical critical parameters, such as chamber design, flow dynamics, and precursor delivery profiles. Consequently, the RP configuration employs a mesh to safeguard the substrate from direct ion bombardment by allowing only neutral radicals to reach the surface, whereas the DP mode allows direct interaction of all plasma species with the substrate surface. As shown in the timing sequence in Figure 1c, the DP-ALD process was conducted with an oxygen injection time of 4 s and a plasma exposure time of 2 s, whereas the RP-ALD process employed an optimized sequence consisting of 10 s oxidation and 15 s plasma generation.

**Figure 2:**
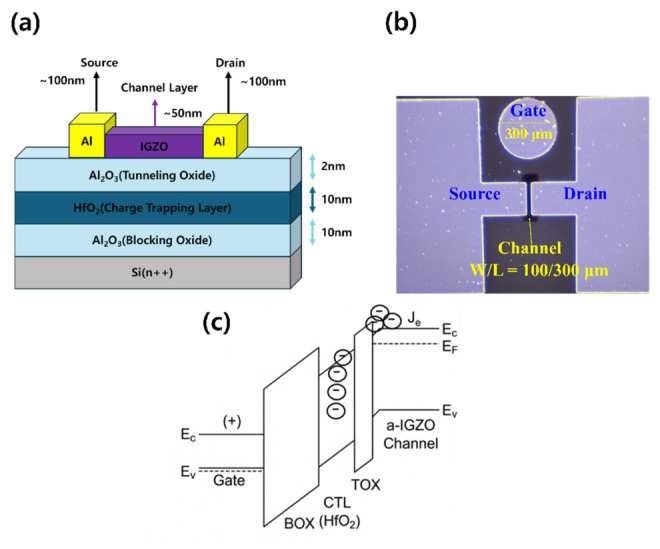
(**a**) Schematic structure of the IGZO flash memory device; (**b**) optical microscopy image of the device; (**c**) energy band diagram illustrating the charge-trapping mechanism.

Bottom-gated flash memory thin-film transistors (TFTs) with an IGZO channel (In:Ga:Zn = 1:1:1 atomic ratio) were fabricated, as shown in Figure 2a. As the control bottom gate of the charge-trapping flash memory device, a heavily doped n-type silicon substrate (resistivity < 0.005 Ω·cm) was employed. The substrate was cleaned using the SC-1 process, followed by a 30 s dilute hydrofluoric acid (HF) dip to remove the native oxide layer. The dielectric stack comprised a blocking layer (Al_2_O_3_, 10 nm), a charge-trapping layer (HfO_2_, 10 nm), and a tunneling layer (Al_2_O_3_, 3 nm), sequentially deposited using both direct plasma ALD (DP-ALD) and remote plasma ALD (RP-ALD). For Al_2_O_3_ deposition, TMA (trimethylaluminum, iChems, Hwaseong, Republic of Korea) was used as the precursor, and the films were deposited at 240 °C in an O_2_ plasma atmosphere with Ar carrier gas (600 sccm) and O_2_ flow (150 sccm). For HfO_2_ deposition, TEMAHf (Tetrakis(ethylmethylamino) hafnium, iChems) was used as the precursor under the same conditions (240 °C, O_2_ plasma ambient).

In the DP-ALD process, plasma directly interacted with the substrate during discharge, whereas in the RP-ALD system, plasma was generated using an inductively coupled plasma method in a region separate from the substrate and then transported via a carrier gas. For a direct comparison under identical conditions, the ALD system (iOV-dx2, iSAC Research, Daejeon, Republic of Korea) was modified to incorporate both DP-ALD and RP-ALD within a single apparatus. In the RP-ALD mode, only neutral plasma radicals could pass through the mesh, whereas in the DP-ALD mode, all the plasma radicals can interact with the substrate. DP-ALD was performed using a built-in plasma generator with O_2_ as the reactive gas and under a plasma power of 150 W, whereas RP-ALD was performed using a remote plasma source (En2ra-RPS, EN2CORE Technology, Daejeon, Republic of Korea) with a plasma power of 2600 W. After deposition of the dielectric stack, a 50-nm-thick amorphous IGZO (a-IGZO) channel layer was deposited using a metal shadow mask. The IGZO channel was deposited by RF magnetron sputtering at room temperature in an Ar atmosphere, with channel dimensions of 100 μm (width) and 300 μm (length). PDA was subsequently performed in a N_2_ ambient at 300, 500, and 700 °C for 20 min to impart the IGZO channel with crystallinity. The source/drain electrodes deposited by DC magnetron sputtering (Sputtering System, BLS, Hwaseong, Republic of Korea) at 100 W for 4 min in an Ar atmosphere. Electrode patterning was achieved using a photolithography process.

Figure 2b presents an optical microscopy (top-view) image of the fabricated IGZO channel device. Figure 2c illustrates a representative energy band diagram of the a-IGZO CTM device in the programming state. As shown, the application of a positive gate voltage induces the injection of electrons from the a-IGZO channel into the HfO_2_ charge-trapping layer through the thin Al_2_O_3_ tunneling oxide. These injected electrons are stored within the HfO_2_ layer, which governs the core operation of the CTM device.

### 2.2. Electrical and Structural Characterization of DP- and RP-ALD Devices

The electrical characteristics of the fabricated three-terminal CTM devices were evaluated using a semiconductor characterization system (4200A-SCS, Keithley, Cleveland, OH, USA) integrated with a microprobe station (APX-6B, WIT Co., Suwon, Republic of Korea). Memory operations were performed within a pulse voltage range of approximately ±15 V and a pulse duration of 1 ms. The electrical characteristics, including charge-trapping capability and reliability, were evaluated for the CTM devices annealed at 300, 500, and 700 °C. Additionally, a UV-erasing process was conducted using a UV LED (*λ* = 395 nm, power = 0.5 W) to reset the memory state by neutralizing trapped charges before each characterization cycle. The structural and morphological properties were analyzed through multistep characterization. The top-view images of the devices were captured using optical microscopy. The thicknesses of the HfO_2_ films deposited using the DP and RP-ALD were measured using spectroscopic ellipsometry (Elli-SE-U, Ellipso Technology, Suwon-si, Republic of Korea). The crystalline structure and interfacial characteristics were confirmed using high-resolution XRD (SmartLab, Rigaku, Tokyo, Japan) and high-resolution transmission electron microscopy (HR-TEM; JEM-2100F (HR), JEOL, Suwon-si, Republic of Korea). The elemental composition of each layer was analyzed via energy-dispersive X-ray spectroscopy (EDS) equipped with the TEM system. Furthermore, the chemical composition and defect states of the HfO_2_ films were analyzed using X-ray photoelectron spectroscopy (Sigma Probe, Thermo Fisher Scientific, Suwon-si, Republic of Korea) with a monochromatic Al Kα source. These comprehensive analyses allowed for the systematic evaluation of the electrical performance and long-term reliability of IGZO-based memory devices.

## 3. Results

### 3.1. Structural and Chemical Characterizations of HfO_2_ Films (DP vs. RP)

**Figure 3:**
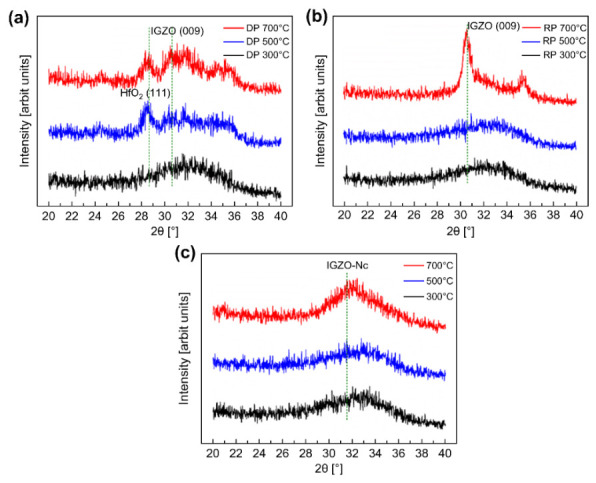
X-ray diffraction (XRD) patterns of (**a**) DP-HfO_2_/IGZO CTM stack; (**b**) RP-HfO_2_/IGZO CTM stack; (**c**) single-layer IGZO film as a function of the post-deposition annealing temperature (300, 500, and 700 °C).

An XRD analysis was performed to investigate the effects of the plasma mode (DP vs. RP) and annealing temperature on the device characteristics. Figure 3 presents the XRD patterns of the HfO_2_/IGZO stacked structure and the single IGZO layer under various thermal conditions. For the samples processed in the direct plasma (DP) mode (Figure 3a), no distinct diffraction peaks were observed at 300 °C, indicating that the film remained in an amorphous state. At 500 °C, a HfO_2_ (111) peak near 28.5° began to emerge, and at 700 °C, both the HfO_2_ (111) peak and a weak IGZO (009) peak near 30.5° were clearly observed. Notably, the crystallization peak of IGZO only emerged at the high temperature of 700 °C in the DP mode.

Figure 3b shows the XRD patterns of the HfO_2_/IGZO thin films deposited using the RP mode. Unlike the DP mode, no distinct HfO_2_ (111) peak was observed at 500 °C, suggesting that the RP-ALD process delays HfO_2_ grain growth in the intermediate temperature range, likely due to the reduced ion bombardment energy during deposition. Under the RP 700 °C condition, a clear and sharp peak was observed at 30.5°, corresponding to the IGZO (009) crystal plane, with a significantly higher intensity compared with the DP 700 °C condition. The presence of this peak indicates that the RP 700 °C process promotes crystalline growth with a strong preferred orientation along the (009) direction.

Figure 3c presents the XRD patterns of the single-layer IGZO film, where only very small peaks can be observed without distinct crystallinity when IGZO exists alone. These results demonstrate that the stacked structure of IGZO and HfO_2_ contributes to enhancing the crystallinity of the IGZO layer, as evidenced by the improved diffraction characteristics within the stack compared with the single-layer film.

**Figure 4:**
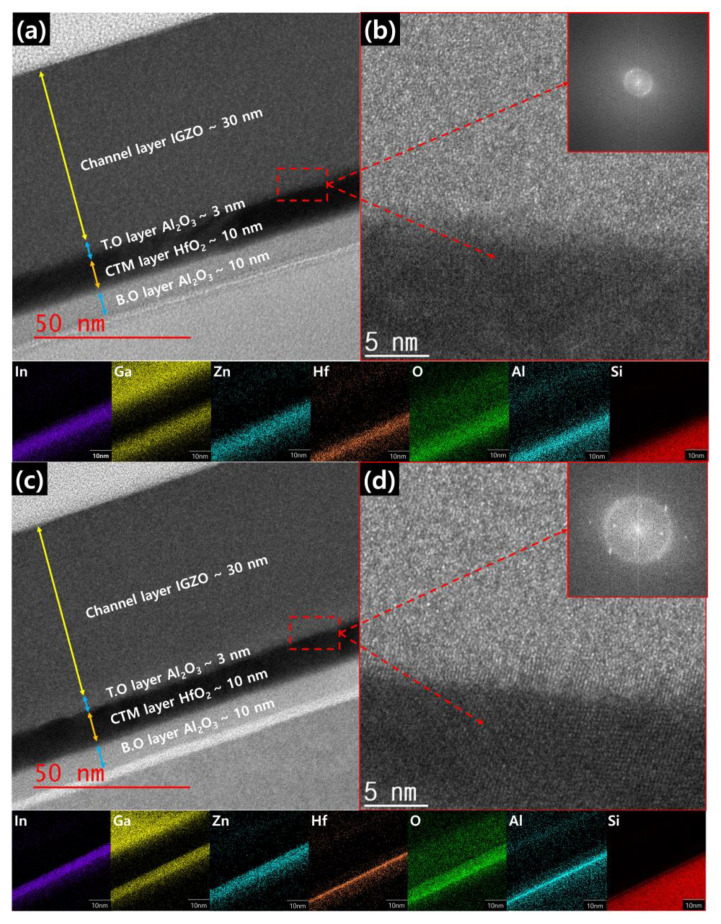
(**a**) HR-TEM image (DP 700 °C), (**b**) IGZO/HfO_2_ interface FFT pattern (DP 700 °C), (**c**) HR-TEM image (RP 700 °C); (**d**) IGZO/HfO_2_ interface FFT pattern (RP 700 °C).

Figure 4 presents the cross-sectional TEM images of the IGZO/HfO_2_ devices annealed at DP 700 °C and RP 700 °C. The DP 700 °C sample was selected based on the preliminary XRD results, which identified it as having the most optimized IGZO diffraction peaks. In contrast, the RP 700 °C sample was chosen to visually verify the c-axis aligned (009) crystallinity previously observed in the RP-mode XRD data.

As shown in Figure 4a,c, both devices exhibit a well-defined vertical stack comprising an IGZO channel layer (~30 nm) and an HfO_2_ charge-trapping layer (~10 nm). The interfaces between the IGZO, HfO_2_, and the adjacent dielectric layers are distinct, with no observable physical defects such as voids or undesirable interfacial reactions. To identify each layer, EDS elemental mapping was performed, and the results are provided below each corresponding image. The EDS analysis confirmed that the In, Ga, and Zn components were clearly distinguished from the Hf and Si elements, demonstrating sharp and well-defined interfaces. For the DP 700 °C device (Figure 4b), the fast Fourier transform (FFT) pattern revealed diffused halo rings along with faint crystalline features, indicating that the IGZO layer maintains a polycrystalline state without a specific preferred orientation. Conversely, the RP 700 °C device (Figure 4d) showed clearly ordered lattice fringes aligned in a specific direction within the IGZO layer. The distinct diffraction spots in the FFT pattern confirmed the formation of crystalline IGZO with a preferred (009) orientation.

**Figure 5:**
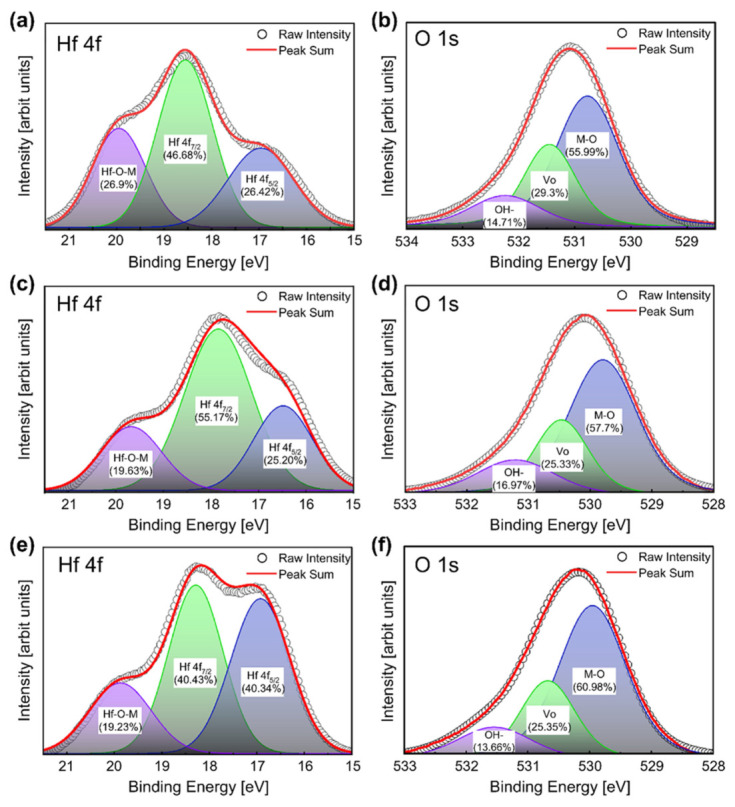
XPS spectra of (**a**,**c**,**e**) Hf 4f and (**b**,**d**,**f**) O 1s core levels for HfO_2_ interface regions for (**a**,**b**) DP 500 °C, (**c**,**d**) RP 500 °C, and (**e**,**f**) RP 700 °C process modes as a function of ALD method and annealing temperature.

The chemical bonding states of the HfO_2_ thin films fabricated via DP- and RP-ALD were compared using XPS as a function of the ALD mode and annealing temperature. Figure 5a,b present the Hf 4f and O 1s spectra of the film deposited by DP-ALD after annealing at 500 °C, while Figure 5c,d show the corresponding spectra of the RP-ALD-deposited film at the same annealing temperature.

A comparison of the Hf 4f spectra reveals that the combined ratio of Hf7/2 and Hf5/2 (corresponding to the stoichiometric Hf4+ state in HfO_2_) was 73.1% for the DP mode, whereas it increased to 80.37% for the RP mode at 500 °C. This indicates a higher concentration of the stoichiometric HfO_2_ phase in the RP-processed film. Additionally, the DP-processed sample exhibited a sub-oxide (Hf–O–M) ratio of 26.9%, which decreased to 19.63% in the RP-processed sample.

This structural improvement is further supported by the O 1s spectral analysis. The ratio of oxygen vacancies (VO) decreased from 29.3% in the DP process to 25.33% in the RP process. Conversely, the ratio of lattice oxygen (M–O), which indicates stronger atomic bonding, increased from 55.99% to 57.7%.

The influence of a higher annealing temperature was further investigated for the RP-ALD process at 700 °C (Figure 5e,f). At 700 °C, the stoichiometric Hf4+ ratio remained consistently high at 80.77%, while the lattice oxygen (M–O) ratio further increased to 60.98%, which was the highest among all conditions. This enhancement suggests that the RP-ALD process not only provides a low-damage deposition environment but also facilitates the formation of a more chemically stable and denser thin film during high-temperature thermal treatment.

These results indicate that the RP-ALD process minimizes plasma-induced damage compared with the DP mode, thereby facilitating the formation of chemically stable thin films with reduced defects and enhanced thermal stability.

### 3.2. Electrical Characteristics of IGZO/HfO_2_ TFTs

**Figure 6:**
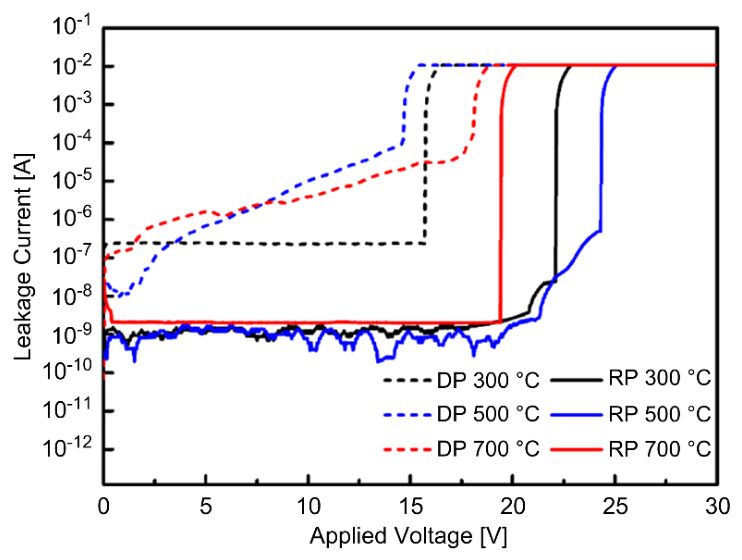
Leakage current density and breakdown voltage (*V_BD_*) characteristics of the HfO_2_/IGZO stacks for DP and RP process modes as a function of annealing temperature (300, 500, and 700 °C).

To evaluate the effects of the plasma mode (DP vs. RP) and annealing temperature on the reliability of gate dielectrics, the leakage current and breakdown voltage (*V*_BD_) characteristics were analyzed using Figure 6. The RP-processed devices (solid lines) exhibited enhanced insulation properties compared with the DP devices (dashed lines). Across all annealing temperatures, the RP samples maintained a stable, low leakage current prior to a distinct hard breakdown. In contrast, the DP samples demonstrated a tendency toward soft breakdown or premature failure, with the leakage current gradually increasing at lower voltages.

The DP 500 °C and DP 700 °C samples exhibited earlier instability, showing an increase in leakage current at applied voltages below approximately 15 V. Conversely, the RP-processed devices recorded higher breakdown voltages. The RP 500 °C condition achieved a maximum *V*_BD_ of approximately 24.5 V. Under the RP 700 °C condition, *V_BD_* decreased slightly to approximately 19.5 V, yet it remained higher than those under all DP conditions.

In summary, the RP process improves the insulation characteristics of the device by minimizing plasma-induced damage and reducing interface defects across the constituent layers [55,56].

**Figure 7:**
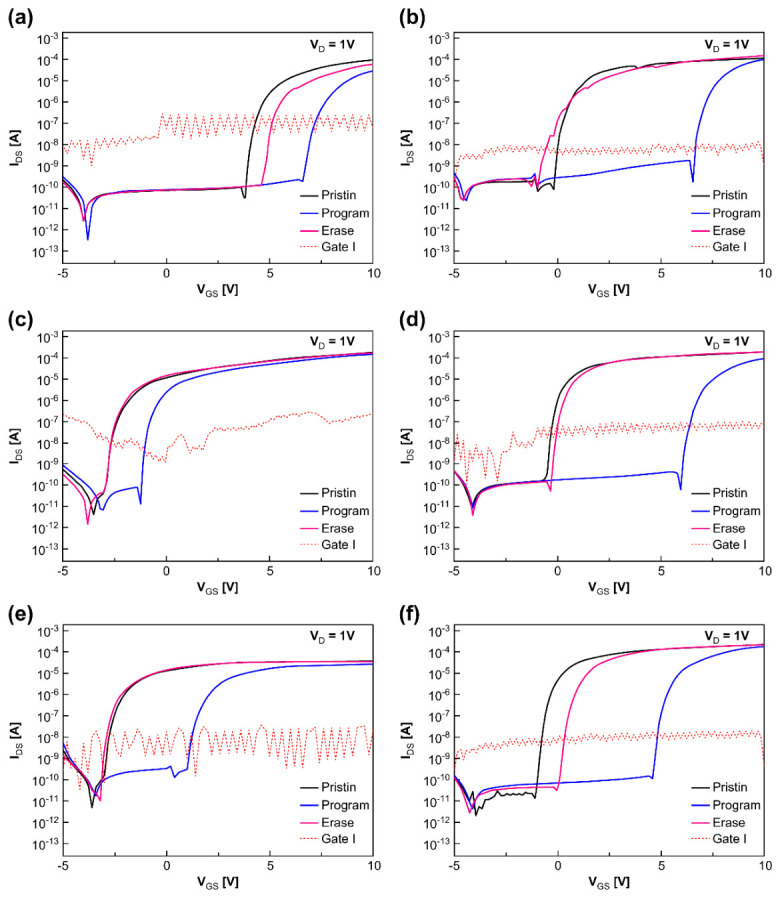
Transfer characteristics under program/erase operations for (**a**,**c**,**e**) direct plasma-processed devices and (**b**,**d**,**f**) remote plasma-processed devices at annealing temperatures of 300, 500, and 700 °C, respectively.

Figure 7 illustrates the transfer characteristics of the fabricated devices in their initial, programmed, and erased states. The programming operation was performed by applying a voltage pulse of 15 V for 1 ms, while the erase operation utilized UV irradiation instead of a negative gate bias. Under these conditions, the DP-processed devices (Figure 7a,c,e) exhibited instability, characterized by a negative shift in the threshold voltage (*V*_th_) during programming and erasing operations.

In contrast, the RP-processed devices (Figure 7b,d,f) demonstrated stable initial *V*_th_ values near 0 V. For CTM operations, the memory window (Δ*V*_th_) improved significantly in the RP devices compared with the DP devices, increasing from 2.0 to 10.4 V at 300 °C, from 2.2 to 9.6 V at 500 °C, and from 4.2 to 7.8 V at 700 °C. Notably, the RP 500 °C condition (Figure 7d) secured a broad memory window while maintaining stable switching behavior.

Consequently, by controlling surface defects and enhancing charge storage performance, the RP process presents potential applicability for realizing future NVM devices [57,58].

**Figure 8:**
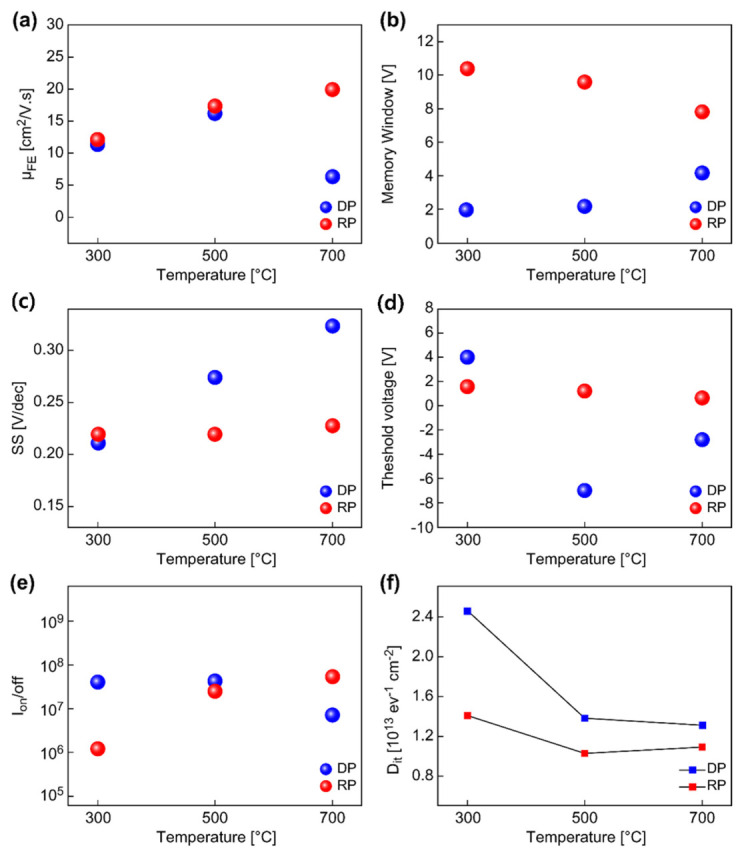
Comparison of electrical parameters extracted from *I_DS_*–*V_GS_* curves: (**a**) Field-effect mobility (*μ_FE_*); (**b**) Memory window (*ΔV_th_*); (**c**) Subthreshold swing (SS); (**d**) Threshold voltage (*Vth*); (**e**) current on/off ratio (*I_on_*/*I_off_*); (**f**) Interface trap density (*D_it_*).

To characterize the electrical properties of the fabricated CTM devices, the field-effect mobility (*μ*_FE_), memory window (Δ*V_t_*_h_), subthreshold swing (SS), threshold voltage (*V*_th_), and interface trap density (*D*_it_) were extracted from the transfer characteristics and are presented in Table 1 and Figure 8. The overall operational characteristics of the RP devices are compared with those of recently reported IGZO-based CTM devices in Table 2.

First, *μ*_FE_ exhibited contrasting trends between the two processes as a function of temperature (Figure 8a). For the RP devices, the mobility increased from 11.95 to 19.8 cm^2^/V·s with an increase in annealing temperature from 300 to 700 °C. In contrast, the DP devices showed an initial increase in mobility up to 500 °C, reaching 16.16 cm^2^/V·s, followed by a decrease to 6.37 cm^2^/V·s at 700 °C.

Second, Δ*V*_th_ and operational stability further distinguished the two processes (Figure 8b,d). Specifically, the RP 500 °C condition demonstrated a balanced performance, achieving a memory window of 9.6 V and the lowest *D*_it_ of 1.03 × 10^13^ eV^−1^ cm^−2^.

The RP devices maintained consistently low SS values ranging from 0.212 to 0.228 V/dec.

**Figure 9:**
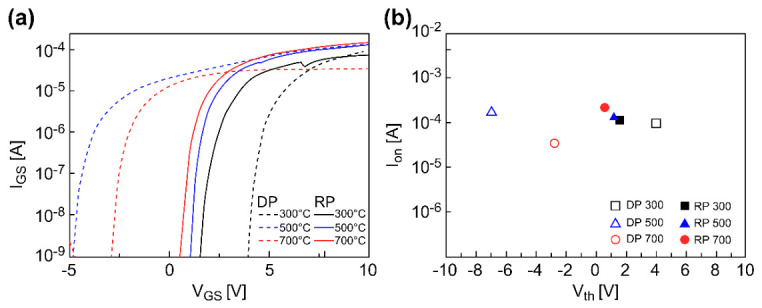
(**a**) Transfer characteristics (*I_DS_*–*V_GS_*) of direct plasma (DP) and remote plasma (RP) devices with varying annealing temperatures (300, 500, and 700 °C); (**b**) *I_on_* vs. *V_th_* distribution map under different process conditions.

To analyze the correlation between the threshold voltage (*V*_th_) variation and electrical performance with respect to the annealing temperature in the DP and RP processes, the variations in the transfer curves and the distributions of key parameters (*I_on_*, *V_th_*) are illustrated in Figure 9.

Figure 9a shows the evolution of the transfer characteristics for the DP and RP devices with an increase in the annealing temperature from 300 to 700 °C. The DP-processed devices (dotted lines) exhibited a shift in the threshold voltage (*V_th_*) and an increased variance with temperature. In contrast, the RP-processed devices (solid lines) demonstrated thermal stability, with the transfer curves remaining largely consistent despite the temperature changes.

Figure 9b presents the *I_on_* and *V_th_* distributions under each process condition. While the parameters for the DP devices are widely dispersed, those for the RP devices are concentrated in the region of high *I_on_* and a *V_th_* value near 0 V. This narrow distribution under the RP conditions indicates a uniform and structurally stable film.

To clarify the discrepancy in the transconductance (*G_m_*), which governs the driving performance of the devices, the channel intrinsic mobility (*μ_0_*), contact resistance (*R_c_*), and interface trap density (*D_it_*) were decoupled and analyzed. For this purpose, the Y-function method was adopted, and each parameter was extracted using the following mathematical derivations.

First, to evaluate the inherent charge transport capability of the channel, *μ*_0_ was extracted using the slope of the Y-function (slope_Y_), as expressed in Equation (1):

(1) $$\mu_{0}=\frac{{\left({slope}_{Y}\right)}^{2}}{\begin{array}{c} \left(W/L\right)\cdot C_{ox}\cdot V_{ds} \end{array}}$$

**Figure 10:**
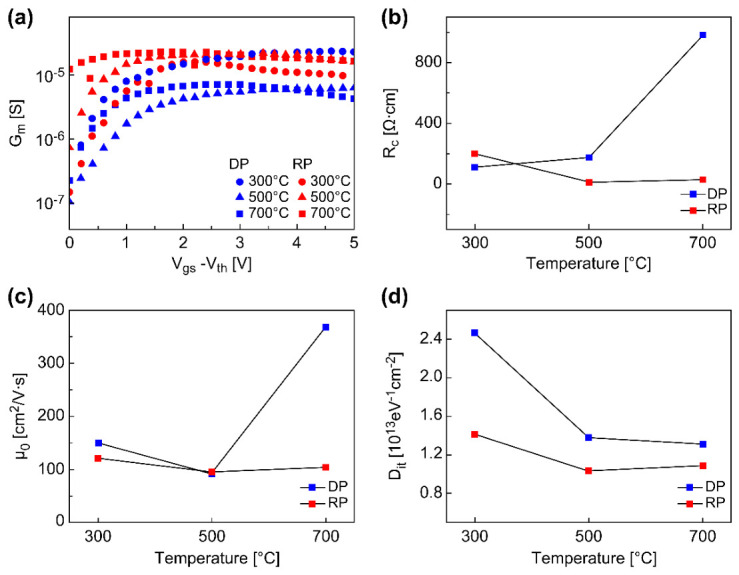
(**a**) Transconductance (*G_m_*) as a function of overdrive voltage (*V_GS_*–*V_th_*); (**b**) contact resistance (*R_c_*); (**c**) intrinsic mobility (*μ*_0_); (**d**) interface trap density (*D_it_*) of direct plasma-processed and remote plasma-processed devices with respect to the annealing temperature.

As illustrated in Figure 10c, the trend in the extracted *μ_0_* contrasts with that observed in *G_m_*. Specifically, a relatively high *μ_0_* was extracted under the DP 700 °C condition, indicating that although crystallization within the channel had progressed, it did not translate into a proportional increase in *G_m_*. This suggests that external factors, rather than the intrinsic channel properties, impeded carrier transport.

To investigate the cause of this discrepancy between *μ_0_* and *G_m_*, the contact resistance (*R_c_*) was calculated based on the difference between the peak transconductance (*g_peak_*) and the theoretical intrinsic transconductance (*g_intrinsic_*), as expressed in Equation (2):

(2) $$R_{c}=\frac{1}{2}(\frac{1}{g_{peak}}-\frac{1}{g_{intrinsic}}),\;\mathrm{where}\;g_{intrinsic}=\frac{W}{L}C_{ox}\mu_{0}V_{ds}$$

Generally, a transfer length method with varying channel lengths is required to precisely determine contact resistance. However, due to the single-device configuration employed in this study, *R_c_* was estimated using Equation (2). The reliability of this extraction is supported by the correlated trends in *μ_0_* (Figure 10c) and *D_it_* (Figure 10d).

As shown in Figure 10b, a significant increase in *R_c_* can be observed under the DP 700 °C condition. This explains the low *G_m_* despite the high *μ_0_*. The resistance generated at the electrode–channel interface (extrinsic), rather than within the channel itself (intrinsic), acts as a primary limiting factor for current flow, thereby degrading overall device performance. In contrast, the RP devices maintained a low *R_c_* across all temperature ranges, allowing the high *μ_0_* to contribute to *G_m_* enhancement without substantial degradation.

Finally, to evaluate the impact of interface quality on switching efficiency, the interface trap density *D_it_* was derived from the SS according to Equation (3):

(3) $$D_{it}=\frac{C_{ox}}{q}(\frac{SS}{{SS}_{ideal}}-1)$$

As illustrated in Figure 10d, the RP devices exhibited lower *D_it_* values overall compared with the DP devices. According to Equation (3), the lower SS values correlate with a reduced *D_it_*, implying that charge loss induced by interface traps is minimized during gate operation.

Consequently, the RP process proves to be an effective approach for realizing high-performance memory devices. By concurrently achieving low *R_c_* and low *D_it_*, the intrinsic performance of the channel is effectively translated into *G_m_* with minimized parasitic loss.

**Figure 11:**
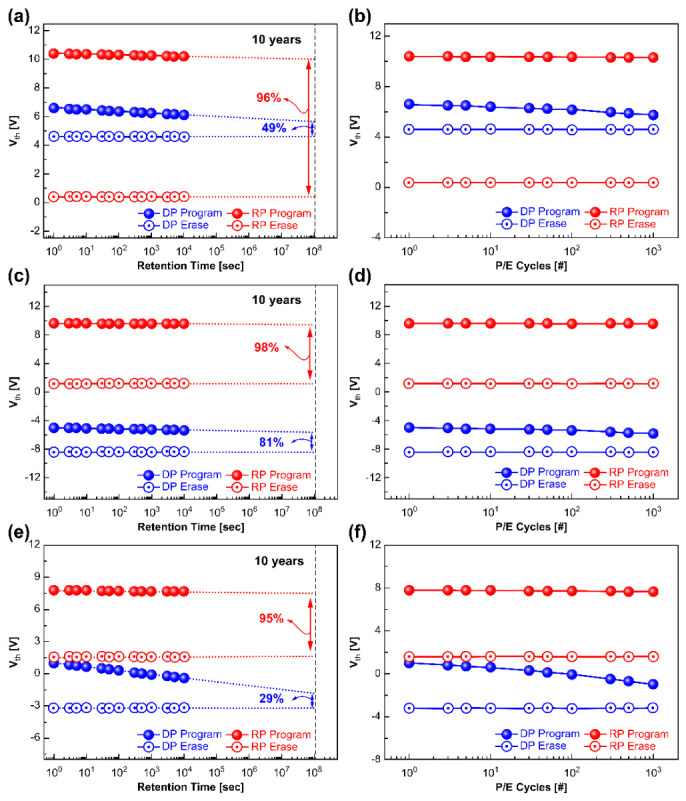
Data retention (**a**,**c**,**e**) and program/erase (P/E) endurance (**b**,**d**,**f**) characteristics of the DP- and RP-processed IGZO/HfO_2_ CTM devices annealed at 300, 500, and 700 °C. The memory states are operated with a programming pulse of 15 V for 1 ms and erased via UV irradiation.

To evaluate the long-term reliability and operational stability of the fabricated CTM devices, data retention and P/E endurance tests were conducted, as shown in Figure 11. For the reliability measurements, the programming operation was performed using a 15 V voltage pulse for 1 ms, and the erase operation was conducted using UV irradiation.

Figure 11a,c,e present the data retention characteristics of the devices extrapolated to 10 years at room temperature. The RP-processed devices exhibited consistent charge retention across all annealing temperatures. Specifically, the RP devices annealed at 300, 500, and 700 °C retained approximately 96%, 98%, and 95% of their initial memory windows after 10 years, respectively. In comparison, the DP-processed devices showed a decrease in charge retention over time, with retention rates of 49%, 81%, and 29% under the 300, 500, and 700 °C conditions, respectively.

Figure 11b,d,f show the P/E endurance characteristics over 10^3^ cycles. The RP-processed devices demonstrated stable cyclic endurance. The RP 500 °C device maintained its initial memory window of approximately 9.6 V with minimal *V_th_* shift throughout the 10^3^ cycles. Conversely, the DP-processed devices displayed a continuous negative shift in the programmed *V_th_*. In particular, the memory window of the DP 700 °C device decreased from its initial 4.2 V as the cycles progressed.

These endurance and retention properties suggest that the HfO_2_/IGZO interface, formed via the combination of RP-ALD and thermal annealing, can trap and maintain electrons while suppressing the generation of stress-induced trap sites.

## 4. Conclusions

Bottom-gate HfO_2_/IGZO CTM devices were fabricated to investigate the effects of the deposition mode (DP-ALD vs. RP-ALD) of the HfO_2_ charge-trapping layer on the crystallization of the overlying IGZO channel and the resulting memory performance.

The analysis revealed that, unlike the DP-ALD process involving high-energy ion bombardment, the RP-ALD process formed a smooth interface without causing physical damage to the HfO_2_ surface. The devices deposited via the RP mode maintained operational stability and exhibited improved performance even after thermal annealing in the range of 300–700 °C.

To decouple the mechanistic effects of these plasma modes on device performance, an electrical analysis using the Y-function method was applied. Although the DP devices exhibited high intrinsic mobility at 700 °C, the actual transconductance decreased due to an increase in contact resistance (*R_c_*) and a high interface trap density (*D_it_*) originating from interface defects. Conversely, the RP devices maintained low *R_c_* and *D_it_* owing to the undamaged interface, which facilitated the translation of the IGZO transport characteristics without significant parasitic loss.

The RP-ALD devices exhibited a broadened memory window (Δ*V_th_*), which is a key performance metric for CTM applications. This enhancement is attributed to the increased charge-trapping efficiency and the suppressed leakage of stored charges achieved by the RP process.

In conclusion, the RP-ALD technique maintains a stable memory window and switching reliability even in high-temperature environments. Therefore, the proposed RP-ALD-based HfO_2_/IGZO structure serves as a reliable approach for securing stable memory characteristics in ALD processes, provided that appropriate plasma processing conditions are adopted.

## Figures and Tables

**Table 1:** Summary of electrical parameters for CTM devices fabricated using DP and RP processes at different annealing temperatures.

Temperature [°C]	Deposition Type	Mobility [cm^2^/V·s]	Memory Window [V]	SS [V/dec]	*V_th_* [V]	*I_on_*/*I_off_*	*D_it_* [10^13^ eV^−1^ cm^−2^]
300	DP	11.46	2	0.211	4	4.04 × 10^7^	2.46
	RP	11.95	10.4	0.212	1.4	8.38 × 10^6^	1.41
500	DP	16.16	2.2	0.274	−7.0	4.31 × 10^7^	1.38
	RP	17.3	9.6	0.219	1.2	2.56 × 10^7^	1.03
700	DP	6.37	4.2	0.324	−2.8	7.19 × 10^6^	1.31
	RP	19.8	7.8	0.228	0.6	5.26 × 10^7^	1.09

**Table 2:** Comparison of memory performance with recent IGZO-based CTM devices.

Channel /Trap Layer	Mobility [cm^2^/V·s]	Memory Window [V]	SS [V/dec]	*I_on_/I_off_*	Reference
IGZO/HfO_2_	11.95	10.4	0.212	8.38 × 10^6^	This Work (RP 300 °C)
IGZO/HfO_2_	17.30	9.6	0.219	2.56 × 10^7^	This Work (RP 500 °C)
IGZO/HfO_2_	19.80	7.8	0.228	5.26 × 10^7^	This Work (RP 700 °C)
IGZO/SiNx	11.6	5.8	0.300	~10^7^	[29]
IGZO/HfO_2_-x	12.0	6.5	0.280	>10^8^	[59]
IGZO/ZnO	7.1	7.2	0.670	~10^7^	[60]

## Data Availability

Data are contained within the article.

## Abbreviations

The following abbreviations are used in this manuscript:

CTM	charge-trapping memory
IGZO	indium gallium zinc oxide
ALD	atomic layer deposition
SCE	short channel effect
RP-ALD	remote plasma ALD
DP-ALD	direct plasma ALD

## References

[1] Burr G.W., Shelby R.M., Sebastian A., Kim S., Kim S., Sidler S., Virwani K., Ishii M., Narayanan P., Fumarola A. (2017). Neuromorphic computing using non-volatile memory. Adv. Phys. X.

[2] Chen A. (2016). A review of emerging non-volatile memory (NVM) technologies and applications. Solid-State Electron..

[3] Ielmini D. (2016). Resistive switching memories based on metal oxides: Mechanisms, reliability and scaling. Semicond. Sci. Technol..

[4] Ielmini D., Wong H.-S.P. (2018). In-memory computing with resistive switching devices. Nat. Electron..

[5] Sze V., Chen Y.-H., Yang T.-J., Emer J.S. (2017). Efficient processing of deep neural networks: A tutorial and survey. Proc. IEEE.

[6] Taur Y., Ning T.H. (2021). Fundamentals of Modern VLSI Devices.

[7] Sze S.M., Ng K.K. (2006). Physics and properties of semiconductors—A review. Physics of Semiconductor Devices.

[8] Meena J.S., Sze S.M., Chand U., Tseng T.-Y. (2014). Overview of emerging nonvolatile memory technologies. Nanoscale Res. Lett..

[9] Goda A. (2021). Recent progress on 3D NAND flash technologies. Electronics.

[10] Banerjee W. (2020). Challenges and applications of emerging nonvolatile memory devices. Electronics.

[11] Zhao C., Zhao C.Z., Taylor S., Chalker P.R. (2014). Review on non-volatile memory with high-k dielectrics: Flash for generation beyond 32 Nm. Materials.

[12] Pan F., Gao S., Chen C., Song C., Zeng F. (2014). Recent progress in resistive random access memories: Materials, switching mechanisms, and performance. Mater. Sci. Eng. R Rep..

[13] Robertson J. (2004). High dielectric constant oxides. Eur. Phys. J. Appl. Phys..

[14] Schroeder U., Hwang C.S., Funakubo H. (2019). Ferroelectricity in Doped Hafnium Oxide: Materials, Properties and Devices.

[15] Müller J., Polakowski P., Mueller S., Mikolajick T. (2015). Ferroelectric hafnium oxide based materials and devices: Assessment of current status and future prospects. ECS J. Solid State Sci. Technol..

[16] Cheema S.S., Kwon D., Shanker N., dos Reis R., Hsu S.-L., Xiao J., Zhang H., Wagner R., Datar A., McCarter M.R. (2020). Enhanced ferroelectricity in ultrathin films grown directly on silicon. Nature.

[17] Cockayne E. (2007). Influence of oxygen vacancies on the dielectric properties of Hafnia: First-principles calculations. Phys. Rev. B.

[18] You H.-W., Cho W.-J. (2010). Charge trapping properties of the HfO_2_ layer with various thicknesses for charge trap flash memory applications. Appl. Phys. Lett..

[19] Xiong K., Robertson J., Gibson M.C., Clark S.J. (2005). Defect energy levels in HfO_2_ high-dielectric-constant gate oxide. Appl. Phys. Lett..

[20] Wilk G.D., Wallace R.M., Anthony J.M. (2001). High-κ gate dielectrics: Current status and materials properties considerations. J. Appl. Phys..

[21] Choi J.H., Mao Y., Chang J.P. (2011). Development of hafnium based high-k materials—A review. Mater. Sci. Eng. R Rep..

[22] Robertson J., Wallace R.M. (2015). High-K Materials and metal gates for CMOS applications. Mater. Sci. Eng. R Rep..

[23] Foster A.S., Lopez Gejo F., Shluger A.L., Nieminen R.M. (2002). Vacancy and interstitial defects in Hafnia. Phys. Rev. B.

[24] Driemeier C., Wallace R.M., Baumvol I.J.R. (2007). Oxygen species in HfO_2_ films: An in situ x-ray photoelectron spectroscopy study. J. Appl. Phys..

[25] Nomura K., Ohta H., Takagi A., Kamiya T., Hirano M., Hosono H. (2004). Room-temperature fabrication of transparent flexible thin-film transistors using amorphous oxide semiconductors. Nature.

[26] Kamiya T., Nomura K., Hosono H. (2010). Present status of amorphous in–Ga–Zn–O thin-film transistors. Sci. Technol. Adv. Mater..

[27] Kamiya T., Hosono H. (2010). Material characteristics and applications of transparent amorphous oxide semiconductors. NPG Asia Mater..

[28] Fortunato E., Barquinha P., Martins R. (2012). Oxide semiconductor thin-film transistors: A review of recent advances. Adv. Mater..

[29] Park E., Woo D.Y., Noh G., Jo Y., Lee D.K., Park J., Kim J., Jeong Y.J., Park S., Jang H.J. (2024). IGZO charge trap flash device for reconfigurable logic functions. Appl. Phys. Lett..

[30] George S.M. (2010). Atomic layer deposition: An overview. Chem. Rev..

[31] Zhang J., Wen X., Hu L., Xu W., Zhu D., Cao P., Liu W., Han S., Liu X., Jia F. (2017). C-axis oriented crystalline IGZO thin-film transistors by magnetron sputtering. J. Mater. Chem. C.

[32] Kim W., Kim J., Ko D., Cha J.-H., Park G., Ahn Y., Lee J.-Y., Sung M., Choi H., Ryu S.W. (2023). Demonstration of crystalline IGZO transistor with high thermal stability for memory applications. Proceedings of the 2023 IEEE Symposium on VLSI Technology and Circuits (VLSI Technology and Circuits).

[33] Chen A., Ma G., Zhang Z., Lin C.-Y., Lin C.-C., Chang T.-C., Tao L., Wang H. (2020). Multi-functional controllable memory devices applied for 3D integration based on a single niobium oxide layer. Adv. Electron. Mater..

[34] Shin Y., Kim S.T., Kim K., Kim M.Y., Oh S., Jeong J.K. (2017). The mobility enhancement of indium gallium zinc oxide transistors via low-temperature crystallization using a tantalum catalytic layer. Sci. Rep..

[35] Kimizuka N., Yamazaki S. (2016). Physics and Technology of Crystalline Oxide Semiconductor CAAC-IGZO: Fundamentals.

[36] Kim H.-M., Kim D.-G., Kim Y.-S., Kim M., Park J.-S. (2023). Atomic layer deposition for nanoscale oxide semiconductor thin film transistors: Review and outlook. Int. J. Extreme Manuf..

[37] Martínez-Puente M.A., Horley P., Aguirre-Tostado F.S., López-Medina J., Borbón-Nuñez H.A., Tiznado H., Susarrey-Arce A., Martínez-Guerra E. (2022). ALD and PEALD deposition of HfO2 and its effects on the nature of oxygen vacancies. Mater. Sci. Eng. B.

[38] Kim K., Oh I.-K., Kim H., Lee Z. (2017). Atomic-scale characterization of plasma-induced damage in plasma-enhanced atomic layer deposition. Appl. Surf. Sci..

[39] Profijt H.B., Potts S.E., van de Sanden M.C.M., Kessels W.M.M. (2011). Plasma-assisted atomic layer deposition: Basics, opportunities, and challenges. J. Vac. Sci. Technol. A.

[40] Price K.M., Najmaei S., Ekuma C.E., Burke R.A., Dubey M., Franklin A.D. (2019). Plasma-enhanced atomic layer deposition of HfO_2_ on monolayer, bilayer, and trilayer MoS_2_ for the integration of high-κ dielectrics in two-dimensional devices. ACS Appl. Nano Mater..

[41] Kim J., Kim S., Jeon H., Cho M.-H., Chung K.-B., Bae C. (2005). Characteristics of HfO_2_ thin films grown by plasma atomic layer deposition. Appl. Phys. Lett..

[42] Knoops H.C.M., Faraz T., Arts K., Kessels W.M.M. (2019). Status and prospects of plasma-assisted atomic layer deposition. J. Vac. Sci. Technol. A.

[43] Xu D., Cheng X., Zhang Y., Wang Z., Xia C., Cao D., Yu Y., Shen D. (2012). Plasma enhanced atomic layer deposition of HfO_2_ with in situ plasma treatment. Microelectron. Eng..

[44] Kuang Y., Zardetto V., van Gils R., Karwal S., Koushik D., Verheijen M.A., Black L.E., Weijtens C., Veenstra S., Andriessen R. (2018). Low-temperature plasma-assisted atomic-layer-deposited SnO_2_ as an electron transport layer in planar perovskite solar cells. ACS Appl. Mater. Interfaces.

[45] Park P.K., Roh J.-S., Choi B.H., Kang S.-W. (2006). Interfacial layer properties of HfO_2_ films formed by plasma-enhanced atomic layer deposition on silicon. Electrochem. Solid-State Lett..

[46] Heil S.B.S., van Hemmen J.L., Hodson C.J., Singh N., Klootwijk J.H., Roozeboom F., van de Sanden M.C.M., Kessels W.M.M. (2007). Deposition of TiN and HfO_2_ in a commercial 200 mm remote plasma atomic layer deposition reactor. J. Vac. Sci. Technol. A.

[47] Park W.-J., Kim H.-J., Lee J.-H., Kim J.-H., Uhm S.-H., Kim S.-W., Lee H.-C. (2024). Characterization of HZO films fabricated by co-plasma atomic layer deposition for ferroelectric memory applications. Nanomaterials.

[48] Lo Nigro R., Schilirò E., Mannino G., Di Franco S., Roccaforte F. (2020). Comparison between thermal and plasma enhanced atomic layer deposition processes for the growth of HfO_2_ dielectric layers. J. Cryst. Growth.

[49] Zhang X.-Y., Yang Y., Zhang Z.-X., Geng X.-P., Hsu C.-H., Wu W.-Y., Lien S.-Y., Zhu W.-Z. (2021). Deposition and characterization of RP-ALD SiO_2_ thin films with different oxygen plasma powers. Nanomaterials.

[50] Van Hemmen H., Heil S., Klootwijk J., Roozeboom F., Hodson C., Van de Sanden R., Kessels E. (2007). Remote plasma and thermal ALD of Al_2_O_3_ for trench capacitor applications. ECS Trans..

[51] Morant C., Galán L., Sanz J.M., An X.P.S. (1990). An XPS Study of the initial stages of oxidation of hafnium. Surf. Interface Anal..

[52] Renault O., Samour D., Damlencourt J.-F., Blin D., Martin F., Marthon S., Barrett N.T., Besson P. (2002). HfO_2_/SiO_2_ interface chemistry studied by synchrotron radiation x-ray photoelectron spectroscopy. Appl. Phys. Lett..

[53] Bouroushian M., Kosanovic T. (2012). Characterization of thin films by low incidence X-ray diffraction. Cryst. Struct. Theory Appl..

[54] Castagné R., Vapaille A. (1971). Description of the SiO_2_-Si interface properties by means of very low frequency MOS capacitance measurements. Surf. Sci..

[55] He G., Zhang L.D., Li G.H., Liu M., Zhu L.Q., Pan S.S., Fang Q. (2005). Spectroscopic ellipsometry characterization of nitrogen-incorporated HfO_2_ gate dielectrics grown by radio-frequency reactive sputtering. Appl. Phys. Lett..

[56] Xiong K., Robertson J. (2005). Point defects in HfO_2_ high K gate oxide. Microelectron. Eng..

[57] Huang L.-T., Chang M.-L., Huang J.-J., Kuo C.-L., Lin H.-C., Liao M.-H., Lee M.-H., Chen M.-J. (2013). Effect of hydrogen participation on the improvement in electrical characteristics of HfO_2_ gate dielectrics by post-deposition remote N_2_, N_2_/H_2_, and NH_3_ plasma treatments. J. Phys. D Appl. Phys..

[58] Abliz A. (2020). Effects of hydrogen plasma treatment on the electrical performances and reliability of InGaZnO thin-film transistors. J. Alloys Compd..

[59] Han J., Jeong B., Sahu D.P., Kim H.M., Yoon T.S. (2023). Non-volatile charge-trap memory characteristics with low-temperature atomic layer deposited HfO_2−x_ charge-trap layer and interfacial tunneling oxide formed by UV/ozone treatment. J. Alloys Compd..

[60] Liu D.D., Pei J., Li L., Huo J., Wu X., Liu W.J., Ding S.J. (2019). Multilevel memory and synaptic characteristics of a-IGZO thin-film transistor with atomic layer-deposited Al_2_O_3_/ZnO/Al_2_O_3_ stack layers. J. Mater. Res..

